# Screening practices, diabetic retinopathy knowledge, and conflict-related barriers to healthcare access among adults with diabetes in Palestine: a cross-sectional study

**DOI:** 10.3389/fcdhc.2026.1814878

**Published:** 2026-04-29

**Authors:** Alhareth M. Amro, Anas K. Assi, Rama F. Rejee, Salahaldeen Deeb, Lama Amro, Zein Abuhantash, Amr Abu Hadwan, Raghad Neiroukh, Shatha Arman, Aya Abufelat, Fatma Qadi, Lamar Aabed, Afnan Manasrah, Abeer Khaleel Srour, Hani Rjoob

**Affiliations:** 1 Faculty of Medicine, Al-Quds University, Jerusalem, Palestine; 2 Faculty of Medicine, Palestine Polytechnic University, Hebron, Palestine; 3 Faculty of Medicine, Mansoura University, Mansoura, Egypt; 4 Faculty of Medicine, Alexandria University, Alexandria, Egypt; 5 Internal Medicine Department, Jericho Governmental Hospital, Jericho, Palestine

**Keywords:** conflict related disruption, diabetes mellitus, diabetic retinopathy, dilated fundus examination, healthcare access, Palestine, retinal screening

## Abstract

**Background:**

Diabetic retinopathy (DR) is a major preventable cause of vision loss. This study assessed DR-related knowledge and screening practices among adults with diabetes in Palestine and explored conflict-related barriers to healthcare access.

**Methods:**

A cross-sectional survey was conducted from June to December 2025 using an Arabic questionnaire distributed in ophthalmic clinics and online. Knowledge was assessed as a continuous score (0–14) and analyzed using multiple linear regression analyses. Comprehensive screening practice was strictly defined as receiving a dilated fundus examination in the preceding 12 months. Multivariable models identified the independent factors associated with knowledge and comprehensive screening.

**Results:**

A total of 740 participants were included (mean age 55.6 years; 59.9% female; 84.2% type 2 diabetes). The mean knowledge score was 10.65/14. Although general awareness was high, disease-specific knowledge and treatment awareness were limited. Multiple linear regression analyses (Adjusted R² = 0.432) demonstrated that higher education, urban residence, and higher income were significantly associated with higher knowledge scores. Notably, reporting conflict-related healthcare disruptions was independently associated with a 1.02-point increase in knowledge (p < 0.001). While 75.0% reported any eye examination in the last year, only 30.7% reported receiving a comprehensive dilated fundus examination. The most common reason for not being examined in the last year was perceiving screening as unnecessary. Conflict-related reduced healthcare access was reported by 67.4%. In multivariable logistic regression analyses, urban residence and a history of eye disease were strong independent correlates of comprehensive screening adherence, whereas the conflict was not significantly associated (p = 0.681).

**Conclusions:**

Despite high general awareness, gaps persist in DR-specific knowledge and in receipt of comprehensive retinal screening. Targeted education, standardized referral pathways, and accessible screening models are needed, particularly for rural and socioeconomically disadvantaged groups where structural and geographic barriers appear to outweigh the acute impact of healthcare disruptions on screening adherence.

## Introduction

Diabetic retinopathy (DR) is a common microvascular consequence of diabetes mellitus (DM) and the primary preventable cause of blindness in the adult population. The restriction of freedom and mobility that come with DM-related visual loss and blindness may severely lower the patients’ quality of life, and shorten their life expectancy (1, 2). Despite its current low incidence, both DR and vision-threatening diabetic retinopathy (VTDR) are predicted to become more common as the prevalence of diabetes rises worldwide (1, 3).

Most of the time, DR is asymptomatic in its early stages. As a result, regular screening, early detection, and timely treatment of DR have been advocated (1, 2). Even though most screening guidelines have different reference standards, a systematic review of the evidence indicates that single-field nonmydriatic photography is the gold standard and the most effective screening method for detecting sight-threatening DR (high sensitivity 87%–97% and specificity 83%–92%). In comparison to direct ophthalmoscopy, it is somewhat cost-effective in screening for a larger population, but it is technically more difficult and more prone to failure due to media opacity (1).

70% to 97% of people with type 1 diabetes and 60% to 80% of people with type 2 diabetes will have some kind of DR after having the condition for more than 15 years. The American Academy of Ophthalmology recommends that patients with type 2 diabetes have their DR evaluated immediately at the time of diagnosis and then every year after that. However, ophthalmoscopy screening is started for type 1 patients five years following diagnosis and every year from then (4).

Longer duration of diabetes, poor blood glucose control, inflammation, hyperlipidemia, obesity, and hypertension are the main risk factors for the development of diabetic retinopathy (DR) in patients with diabetes (2). DR can be managed in a number of ways, such as primary prevention, which delays the onset of DR in diabetic patients, secondary prevention, which halts the progression of DR, and tertiary prevention, which uses intravitreal anti-vascular endothelial growth factor therapy (anti-VEGF) injections, laser photocoagulation, and vitreoretinal surgery to treat complications (5). It has been demonstrated that strict glycemic control with insulin and oral hypoglycemic medications alone greatly lowers the risk of retinopathy and its advancement. Insulin therapy, which is mostly used to treat type I diabetes, has been demonstrated to have a local effect on ocular tissue through the restoration of rod photoreceptor function and the retinal insulin receptor signaling cascade (1).

A number of factors have been linked to patients’ poor adherence to routine eye exams, including younger age, lower income, lower education, race, insulin use, fewer comorbidities, less diabetes education, lower adherence to oral hypoglycemic agents, hemoglobin A1C >9%, cost, lack of insurance, inability to get a timely diabetic foot examination, living in a rural area, and not having easy access to the closest ophthalmologist (1, 5). Identifying factors related to non-compliance with retinal screening procedures among individuals with diabetes may help policymakers develop targeted strategies to improve adherence and, in turn, reduce the resulting visual impairment and disease burden (2, 5). High socioeconomic position, higher educational attainment, longer duration of diabetes, and female sex were all positively correlated with having better understanding of DR and regular eye exams (6).

In Palestine, where diabetes and diabetic retinopathy represent an important public health concern and where access to routine healthcare may be disrupted by conflict-related constraints, understanding both patient knowledge and actual retinal screening behavior is essential for designing effective prevention strategies. Although previous studies have examined awareness and eye-care practices in other settings, evidence from Palestine remains limited, particularly regarding the distinction between general eye examination and comprehensive diabetic retinopathy screening, as well as the role of conflict-related barriers to healthcare access. The Aim of this study was to assess diabetic retinopathy-related knowledge, screening practices, and conflict-related barriers to healthcare access among adults with diabetes in Palestine, and to identify factors associated with knowledge and comprehensive screening adherence.

## Methodology

### Study design and setting

This cross-sectional study was conducted in Palestine between June 2025 and December 2025 among adults with diabetes mellitus. Data were collected using a hybrid recruitment strategy comprising clinic-based recruitment and online survey dissemination. Clinic-based recruitment was conducted in four ophthalmology/eye-care clinics located in Hebron, Bethlehem, Ramallah and Jerusalem. These facilities were selected based on accessibility, patient volume, institutional collaboration, geographic coverage and feasibility. Clinic-based recruitment was undertaken in ophthalmology clinics because the study specifically focused on diabetic retinopathy knowledge and retinal screening practices, and these clinics provided access to individuals with diabetes who were able to report on eye-care experiences. The online component was implemented to increase reach beyond facility attendees and to include adults with diabetes from different areas of Palestine.

### Inclusion and exclusion criteria

Eligible participants were Palestinian adults aged 18 years or older with self-reported diabetes mellitus who were able to complete the questionnaire independently in Arabic. Individuals with cognitive impairment or those unable to complete the survey independently were excluded. A total of 740 respondents met the eligibility criteria and were included in the final analysis.

### Sample size calculation

The minimum required sample size was estimated *a priori* using the Raosoft sample size calculator. The calculation was based on the primary descriptive outcome of comprehensive screening adherence and assumed an expected prevalence of 50%, a 95% confidence level, and a 5% margin of error. The 50% assumption was used in the absence of robust national prevalence estimates for this outcome and was selected because it provides the most conservative sample size estimate. This yielded a minimum required sample of 384 participants. The final sample of 740 respondents exceeded this minimum, thereby improving statistical precision and analytical power.

### Questionnaire instrument

We adapted a structured questionnaire from the previously published instrument by Assem et al. assessing diabetic retinopathy-related knowledge and eye-care practices (7). The questionnaire was translated from English into Arabic by two bilingual medical experts, and discrepancies were resolved by consensus to ensure conceptual equivalence and cultural appropriateness. Content and face validity were reviewed before administration. A pilot study involving 30 participants from the target population was conducted to assess clarity and comprehensibility; no major modifications were required, and pilot responses were excluded from the final analysis.

The final questionnaire consisted of four sections. The first section collected sociodemographic and clinical information, including age, sex, place of residence, educational level, occupation, monthly income, self-reported type of diabetes, and disease duration. The second section assessed diabetic retinopathy knowledge across 14 scored knowledge components covering risk factors, complications, screening recommendations, and treatment options. Each correct response received one point, whereas incorrect and “do not know” responses received zero points. A composite knowledge score ranging from 0 to 14 was calculated and retained as a continuous variable for the primary analysis to avoid the loss of information associated with arbitrary dichotomization. The internal consistency of the knowledge scale was acceptable (Cronbach’s alpha = 0.738). The third section assessed practices related to diabetic retinopathy screening. For analytical purposes, comprehensive screening adherence was defined *a priori* as self-reported receipt of a dilated fundus examination within the preceding 12 months. The fourth section assessed conflict-related barriers to healthcare access and changes in health-monitoring behavior during the conflict period.

### Data collection procedure

Data collection was conducted using two distinct approaches:

#### Clinic-based recruitment

In participating ophthalmology clinics, potentially eligible patients attending the clinic during the data collection period were approached using convenience sampling. QR codes linking to the Google Forms questionnaire were displayed in waiting areas, allowing interested patients to complete the survey on their mobile devices while attending the clinic.

#### Online recruitment

The same questionnaire link was disseminated through diabetes-related Facebook pages and WhatsApp groups using a snowball sampling approach. Participants were encouraged to share the survey link with other adults with diabetes in their networks.

The questionnaire was self-administered in Arabic, required approximately 10 minutes to complete, and participation was voluntary, anonymous, and without incentives. Before accessing the questionnaire, respondents were presented with an introductory page describing the study objectives, procedures, confidentiality safeguards, and their right to decline participation. Responses were recorded automatically and stored in a secure electronic database accessible only to the research team.

### Data analysis

We exported completed responses from Google Forms into SPSS version 25 for analysis. We checked data for completeness and consistency before analysis. We summarized categorical variables using frequencies and percentages. We summarized continuous variables using means and standard deviations. To identify factors independently associated with the study outcomes, we employed two distinct multivariable modeling approaches. First, a multiple linear regression model was conducted to identify factors associated with the continuous knowledge score (0–14). The overall model fit was evaluated using the adjusted R-squared, and results were reported as unstandardized coefficients (β) with 95 percent confidence intervals. Second, a multivariable binary logistic regression model was utilized to identify factors associated with comprehensive screening practice (dilated fundus examination). Results were reported as adjusted odds ratios with 95 percent confidence intervals, and model fit was confirmed using the Hosmer-Lemeshow goodness-of-fit test.

Covariates for both models were initially selected based on clinical relevance and bivariable significance. Crucially, prior to finalizing the multivariable models, multicollinearity was rigorously assessed using the Variance Inflation Factor (VIF). Variables demonstrating severe collinearity (such as occupation overlapping with sex) or possessing excessively small sub-categories (such as specific diabetes types) were excluded to ensure mathematical stability, confirming all retained variables had a VIF < 5. Furthermore, the primary conflict-related variable (impact of war on healthcare access) was forced into both final multivariable models to evaluate its independent impact on health literacy and screening adherence. We considered a p-value less than 0.05 statistically significant and used two-sided tests throughout.

### Handling of missing data

Given the online design, responses to knowledge and practice items were mandatory, which minimized missing data for primary outcomes. For sociodemographic and clinical variables, we excluded cases with more than 10 percent missing responses from the analysis. Cases with incidental missing data on remaining variables were handled using complete case analysis (listwise deletion), as the proportion of missingness was minimal.

### Ethical considerations

Ethical approval for this study was provided by the Research Ethical Committee at Al-Quds University (Ref no. 601/REC/2025). All procedures complied with the ethical principles of the Declaration of Helsinki. Before starting the questionnaire, participants reviewed an electronic informed consent statement detailing the study objectives, voluntary nature of participation, confidentiality measures, and the right to withdraw at any time. Only participants who provided consent were allowed to proceed. We collected no identifying information, and we stored all data securely with access limited to the research team.

## Results

### Socio-demographic and clinical characteristics

A total of 740 participants diagnosed with diabetes mellitus (DM) were included in the final analysis. The study population exhibited a female predominance, with 443 (59.9%) females and 297 (40.1%) males. The mean age of the participants was 55.6 ± 14.1 years. Regarding residential distribution, nearly two-thirds of the participants (n=473, 63.9%) resided in urban areas, while 267 (36.1%) lived in rural settings. The majority of participants were married (80.5%). Educational attainment varied, with 39.1% (n=289) holding a tertiary (college or university) degree, whereas 17.0% (n=126) had only completed primary education. In terms of economic status, 71.6% (n=530) of families reported a middle monthly income sufficient for basic needs, while 23.8% (n=176) reported a low income that did not suffice for basic needs. Socio-demographic characteristics are detailed in **Table 1**.

**Table 1:** Sociodemographic characteristics of study participants (N = 740).

Characteristic	Total, n (%)
Sex	
Male	297 (40.1)
Female	443 (59.9)
Age (years), mean ± SD	55.6 ± 14.1
Residence	
Urban	473 (63.9)
Rural	267 (36.1)
Marital Status	
Single	67 (9.1)
Married	596 (80.5)
Divorced	8 (1.1)
Widowed	69 (9.3)
Educational Level	
Primary (5–9 years)	126 (17.0)
Preparatory (10–12 years)	124 (16.8)
Secondary (13–17 years)	201 (27.2)
Tertiary (College/University) (18 – 24 years)	289 (39.1)
Occupation	
Daily laborer	60 (8.1)
Government employed	133 (18.0)
House wife	387 (52.3)
Merchant	31 (4.2)
Retired	62 (8.4)
Other	67 (9.1)
Family Monthly Income	
Low (does not suffice for basic needs)	176 (23.8)
Middle (suffices for basic needs)	530 (71.6)
High (suffices for basic needs and comfort)	34 (4.6)

SD, standard deviation.

Clinically, the vast majority of participants were diagnosed with Type 2 Diabetes Mellitus (84.2%, n=623), with Type 1 Diabetes Mellitus accounting for 11.4% (n=84). A notable proportion (40.4%, n=299) had a disease duration exceeding 10 years, and 20.9% (n=155) had a duration between 5 and 10 years. Comorbid hypertension was highly prevalent, affecting 65.8% (n=487) of the study population. Additionally, 62.2% (n=460) of participants reported a history of eye disease, while 34.9% (n=258) reported no such history. Clinical characteristics are detailed in **Table 2**.

**Table 2:** Clinical characteristics of study participants (N = 740).

Variable	Total, n (%)
Type of Diabetes Mellitus	
Type 1	84 (11.4)
Type 2	623 (84.2)
Do not know	33 (4.5)
Duration of Diabetes (years)	
< 1	85 (11.5)
1–5	201 (27.2)
5–10	155 (20.9)
> 10	299 (40.4)
Hypertension	
Yes	487 (65.8)
No	240 (32.4)
Do not know	13 (1.8)
History of Eye Disease	
Yes	460 (62.2)
No	258 (34.9)
Do not know	22 (3.0)

### Knowledge of diabetic retinopathy

The assessment of diabetic retinopathy (DR) knowledge revealed a clear gap between general awareness and disease-specific understanding. While 94.9% of participants correctly recognized that diabetes can affect the eyes and 88.0% knew that diabetes can cause blindness, only 54.3% correctly identified diabetic retinopathy as the specific eye condition caused by diabetes. In addition, only 42.6% correctly identified DR as involving changes in the retinal blood vessels, whereas 41.2% reported that they did not know the nature of the condition (**Table 3**). Regarding risk factors for diabetic retinopathy, awareness of poor glycemic control was high, with 84.1% identifying poorly controlled blood sugar as a risk factor and 94.2% acknowledging the importance of blood sugar control in preventing blindness. By contrast, awareness of other established risk factors was substantially lower, with 53.9% identifying longer duration of diabetes, 53.8% identifying hypertension, and only 12.6% identifying smoking as a risk factor. Knowledge of treatment options was also limited, as 32.2% of participants did not know any available treatment modality, while 25.1% identified intravitreal injections and 22.0% identified laser photocoagulation (**Table 3**). Although 95.7% of respondents believed that people with diabetes require eye screening, only 69.1% stated that ophthalmic evaluation should occur immediately after diagnosis. The mean total knowledge score was 10.65 ± 2.93 out of 14 (**Table 3**).

**Table 3:** Knowledge of diabetic retinopathy among participants with diabetes (N = 740).

Question/item	n	%
Does diabetes affect the eye?		
Yes	702	94.9
No	21	2.8
I do not know	17	2.3
Can diabetes cause blindness?		
Yes	651	88.0
No	18	2.4
I do not know	71	9.6
What eye condition does diabetes specifically cause?		
Diabetic retinopathy (correct)	402	54.3
Glaucoma	105	14.2
Other	13	1.8
I do not know	220	29.7
What is diabetic retinopathy?		
Changes in blood vessels of the retina (correct)	315	42.6
High sugars in the eye	66	8.9
Same as cataract	36	4.9
High pressure in the eye	18	2.4
I do not know	305	41.2
Risk factors for diabetic retinopathy*		
Poorly controlled blood sugar	622	84.1
Duration of diabetes	399	53.9
Hypertension	398	53.8
High BMI	314	42.4
Pregnancy	51	6.9
Smoking	93	12.6
Psychological factors	209	28.2
I do not know	78	10.5
Should a person with diabetes check blood pressure?		
Yes	683	92.3
No	28	3.8
I do not know	29	3.9
Is blood sugar control important in preventing blindness?		
Yes	697	94.2
No	7	0.9
I do not know	36	4.9
Is diabetic eye disease treatable?		
Yes	542	73.2
No	53	7.2
I do not know	145	19.6
Treatment options for diabetic eye disease		
Laser photocoagulation	163	22.0
Intravitreal injections	186	25.1
Surgery	153	20.7
I do not know	238	32.2
Should a person with diabetes receive eye screening?		
Yes	708	95.7
No	7	0.9
I do not know	25	3.4
How soon after diagnosis should a person visit an ophthalmologist?		
Immediately after diagnosis	511	69.1
One year after diagnosis	73	9.9
Five years after diagnosis	16	2.2
Other	140	18.9
Does a person with diabetes need regular eye examination?		
Yes	683	92.3
No	13	1.8
I do not know	44	5.9
Total Knowledge Score (out of 14 points)		
Mean ± SD	10.65 ± 2.93	
Median (IQR)	11.0 (9.0–14.0)	
Range	0–14	

* Multiple-response item; percentages do not sum to 100% because participants could select more than one option. Denominator for percentage calculations: N = 740.

### Screening practices and attitudes

Regarding eye-care practices, 73.2% of participants reported ever being referred to an ophthalmologist, and 81.9% reported having undergone an eye examination at least once since their diabetes diagnosis. In the preceding 12 months, 37.4% reported three or more eye examinations, 21.2% reported one examination, and 16.4% reported two examinations, whereas 25.0% reported no eye examination during that period (**Table 4**). However, the type of examination received varied considerably. Only 30.7% of participants reported having undergone a dilated fundus examination, which was the study definition of comprehensive screening adherence. By contrast, 35.1% reported vision examination alone, highlighting a potential misconception between routine visual assessment and appropriate retinal screening for diabetic retinopathy (**Table 4**). Among participants who did not undergo eye examination in the preceding year, the most frequently reported reason was the perception that screening was unnecessary, followed by lack of physician advice and financial constraints (**Table 4**).

**Table 4:** Eye-care practices and attitudes among participants with diabetes (N = 740).

Question/item	n	%
How did you come to know about diabetes affecting the eyes?		
Health professional at the diabetes follow-up clinic	325	43.9
Eye-care professional	173	23.4
Family member/relative/friend with diabetes	155	20.9
TV, magazines, or other media	56	7.6
Did not know	31	4.2
Have you ever been referred to see an ophthalmologist?		
Yes	542	73.2
No	191	25.8
I do not know	7	0.9
Have your eyes been examined by an ophthalmologist after diagnosis of diabetes?		
Yes	606	81.9
No	124	16.8
I do not know	10	1.4
If yes, how many times in the last year?		
Three times or more	277	37.4
Once	157	21.2
Twice	121	16.4
None	49	6.6
Did not examine	136	18.4
What kind of eye examination do you get?		
Vision examination	260	35.1
Dilated examination of the back of the eye	227	30.7
Did not examine	166	22.4
Slit-lamp/torch examination	59	8.0
Other	18	2.4
Checking for eyeglasses	10	1.4
If you did not undergo an eye examination, what was the main reason?		
I did not think it was necessary	112	15.1
I was not advised to by my doctor	36	4.9
Financial restrictions	26	3.5
Lack of convenient facility	10	1.4
Not applicable (did examine)	556	75.1
Do you check your blood sugar?		
Yes	692	93.5
No	39	5.3
I do not know	5	0.7
Other	4	0.5
What was your latest HbA1c?		
Less than 6.5	80	10.8
6.5–7.5	142	19.2
7.5–8.5	249	33.6
8.5–9.5	89	12.0
9.5–10.5	86	11.6
More than 10.5	60	8.1
I do not know	34	4.6

### Impact of conflict on healthcare access

The study further evaluated the collateral impact of the Gaza conflict (commencing October 7) on healthcare accessibility and patient behavior regarding eye care. A significant majority of participants (67.4%, n=499) reported that the conflict adversely affected their ability to access general healthcare services. When characterizing the specific impediments faced, economic instability emerged as the predominant barrier, with 40.0% (n=296) citing income loss or unemployment, closely followed by logistical constraints related to movement restrictions and military checkpoints (38.4%, n=284). Disruption of medical infrastructure was also noted, with 19.2% (n=142) reporting difficulties due to the closure or dysfunction of health centers. Despite these systemic challenges, resilience in specific eye care practices was observed; 67.6% (n=500) of patients maintained their regular eye examinations during the conflict period. Among the 32.4% (n=240) who were unable to undergo examinations, the primary deterrent was a shift in prioritization rather than a direct lack of access; 21.6% (n=160) indicated they did not consider eye care a priority amidst the conflict, while smaller proportions cited financial incapacity (5.8%) or preoccupation with conflict circumstances (3.2%). Interestingly, the conflict appeared to positively influence health-seeking attitudes for a plurality of patients, as nearly half of the participants (46.6%, n=345) reported a heightened desire to monitor their health status following the onset of the conflict. Conflict-related impacts are summarized in **Table 5**. These descriptive attitudes were statistically corroborated in our multivariable analyses. Reporting conflict-related healthcare access barriers was independently associated with a significant increase in the DR knowledge score (β = 1.02, p < 0.001, **Table 6**). Conversely, the conflict was not independently associated with actual comprehensive screening adherence (AOR = 1.09; 95% CI: 0.72–1.66; p = 0.680, **Table 7**), indicating a critical divergence between conflict-induced health awareness and the physical ability to overcome underlying geographic and structural barriers to receive a dilated exam.

**Table 5:** Conflict-related barriers to healthcare access and eye care (N = 740).

Question/item	n	%
Did the Gaza war (7 October 2023) affect your ability to access healthcare services?		
Yes	499	67.4
No	241	32.6
Types of difficulties faced*		
Financial reasons (income loss/unemployment)	296	40.0
Difficulty in movement/military checkpoints	284	38.4
No difficulties faced	239	32.3
Closure or disruption of health centers	142	19.2
Other priorities (food, shelter, security)	35	4.7
During the conflict period, were you able to perform regular eye examinations?		
Yes	500	67.6
No	240	32.4
If unable to examine, what was the main reason?		
Did not consider it a priority	160	21.6
Weak financial capabilities	43	5.8
Preoccupied with war circumstances	24	3.2
Lack of medical facilities	19	2.6
Not applicable (was able to examine)	494	66.8
After the war, did your desire to monitor your health status increase?		
Yes	345	46.6
No	178	24.1
I do not know	217	29.3

*Multiple-response item; percentages do not sum to 100% because participants could select more than one option. Denominator for percentage calculations: N = 740.

**Table 6:** Factors associated with knowledge score of diabetic retinopathy: multivariable linear regression analysis (N = 740).

Variable	Category	Mean knowledge score (± SD)	β	95% CI	p-value
(Intercept)	—	—	12.43	(11.43 to 13.43)	<0.001
Age (years)	Continuous	55.5 ± 14.1	0.003	(-0.012 to 0.018)	0.707
Sex					
Female	Reference	10.4 ± 2.8	0.00 (Ref)	—	—
Male		11.0 ± 2.6	-0.01	(-0.35 to 0.34)	0.966
Residence					
Urban	Reference	11.2 ± 2.4	0.00 (Ref)	—	—
Rural		9.6 ± 3.1	-0.87	(-1.27 to -0.48)	<0.001*
Marital Status					
Married	Reference	10.8 ± 2.7	0.00 (Ref)	—	—
Single		10.1 ± 3.1	-1.18	(-1.83 to -0.53)	<0.001*
Divorced		12.0 ± 2.4	-0.30	(-1.86 to 1.27)	0.710
Widowed		9.8 ± 3.1	-0.52	(-1.12 to 0.09)	0.093
Educational Level					
Tertiary (university)	Reference	12.0 ± 2.0	0.00 (Ref)	—	—
Secondary		10.5 ± 2.7	-0.89	(-1.31 to -0.47)	<0.001*
Preparatory		9.2 ± 3.0	-2.20	(-2.73 to -1.68)	<0.001*
Primary		9.1 ± 2.9	-2.43	(-3.00 to -1.86)	<0.001*
Family Monthly Income					
Middle (suffices)	Reference	11.1 ± 2.5	0.00 (Ref)	—	—
High (suffices/comfort)		10.6 ± 2.7	0.10	(-0.71 to 0.90)	0.811
Low (insufficient)		9.4 ± 3.1	-1.02	(-1.48 to -0.57)	<0.001*
Diabetes Duration					
1–5 years	Reference	11.0 ± 2.5	0.00 (Ref)	—	—
<1 year		11.1 ± 2.6	-0.002	(-0.57 to 0.57)	0.994
5–10 years		10.4 ± 2.8	0.02	(-0.46 to 0.49)	0.939
>10 years		10.3 ± 3.0	-0.37	(-0.80 to 0.05)	0.084
Hypertension					
Yes	Reference	10.9 ± 2.6	0.00 (Ref)	—	—
No		10.1 ± 3.1	-0.74	(-1.14 to -0.33)	<0.001*
Unknown		11.8 ± 1.9	-1.05	(-2.32 to 0.21)	0.103
Previous Eye Disease^†^					
Yes	Reference	11.2 ± 2.3	0.00 (Ref)	—	—
No		9.7 ± 3.2	-1.25	(-1.64 to -0.86)	<0.001*
Unknown		10.4 ± 2.4	-1.57	(-2.56 to -0.59)	0.002*
War Affected Access					
No	Reference	9.8 ± 3.2	0.00 (Ref)	—	—
Yes		11.1 ± 2.4	1.02	(0.64 to 1.40)	<0.001*

Model statistics: Adjusted R² = 0.432; F (19, 720) = 30.56, p < 0.001. Abbreviations: β, unstandardized coefficient; CI, confidence interval; SD, standard deviation. ^†^History of eye disease in the past year. *Indicates statistical significance at p < 0.05. Occupation and type of diabetes were excluded due to multicollinearity (VIF > 5).

**Table 7:** Factors associated with comprehensive screening adherence: multivariable logistic regression analysis (N = 740).

Variable	Category	No dilated exam, n (%)	Dilated exam, n (%)	Crude OR (95% CI)	p-value	Adjusted OR (95% CI)^‡^	p-value
Age (years)	Continuous	—	—	—	—	1.01 (1.00–1.03)	0.180
Sex							
Female	Reference	324 (63.2)	119 (52.4)	1.00 (Reference)	—	1.00 (Reference)	—
Male		189 (36.8)	108 (47.6)	1.56 (1.13–2.14)	0.010*	1.33 (0.94–1.90)	0.110
Residence							
Urban	Reference	289 (56.3)	184 (81.1)	1.00 (Reference)	—	1.00 (Reference)	—
Rural		224 (43.7)	43 (18.9)	0.30 (0.21–0.44)	<0.001*	0.42 (0.27–0.66)	<0.001*
Marital Status							
Married	Reference	400 (78.0)	196 (86.3)	1.00 (Reference)	—	1.00 (Reference)	—
Single		54 (10.5)	13 (5.7)	0.49 (0.25–0.89)	0.030*	0.65 (0.30–1.32)	0.240
Divorced		5 (1.0)	3 (1.3)	1.22 (0.25–5.04)	0.780	1.03 (0.20–4.70)	0.960
Widowed		54 (10.5)	15 (6.6)	0.57 (0.30–1.01)	0.060	0.76 (0.38–1.49)	0.440
Educational Level							
Tertiary (university)	Reference	174 (33.9)	115 (50.7)	1.00 (Reference)	—	1.00 (Reference)	—
Secondary		140 (27.3)	61 (26.9)	0.66 (0.45–0.96)	0.030*	0.91 (0.59–1.38)	0.640
Preparatory		95 (18.5)	29 (12.8)	0.46 (0.28–0.74)	<0.001*	0.64 (0.36–1.11)	0.120
Primary		104 (20.3)	22 (9.7)	0.32 (0.19–0.53)	<0.001*	0.46 (0.24–0.86)	0.020*
Family Monthly Income							
Middle (suffices)	Reference	343 (66.9)	187 (82.4)	1.00 (Reference)	—	1.00 (Reference)	—
High (suffices)		27 (5.3)	7 (3.1)	0.48 (0.19–1.06)	0.090	0.64 (0.24–1.53)	0.340
Low (insufficient)		143 (27.9)	33 (14.5)	0.42 (0.27–0.64)	<0.001*	0.82 (0.48–1.37)	0.450
Diabetes Duration							
1–5 years	Reference	140 (27.3)	61 (26.9)	1.00 (Reference)	—	1.00 (Reference)	—
<1 year		57 (11.1)	28 (12.3)	1.13 (0.65–1.93)	0.670	1.08 (0.60–1.94)	0.790
5–10 years		120 (23.4)	35 (15.4)	0.67 (0.41–1.08)	0.100	0.80 (0.47–1.34)	0.400
>10 years		196 (38.2)	103 (45.4)	1.21 (0.82–1.78)	0.340	1.46 (0.94–2.27)	0.090
Hypertension							
Yes	Reference	316 (61.6)	171 (75.3)	1.00 (Reference)	—	1.00 (Reference)	—
No		191 (37.2)	49 (21.6)	0.47 (0.33–0.68)	<0.001*	0.78 (0.50–1.21)	0.270
Unknown		6 (1.2)	7 (3.1)	2.16 (0.71–6.79)	0.170	3.03 (0.85–11.40)	0.090
Previous Eye Disease^†^							
Yes	Reference	289 (56.3)	171 (75.3)	1.00 (Reference)	—	1.00 (Reference)	—
No		208 (40.5)	50 (22.0)	0.41 (0.28–0.58)	<0.001*	0.56 (0.36–0.85)	0.010*
Unknown		16 (3.1)	6 (2.6)	0.63 (0.22–1.57)	0.350	0.54 (0.18–1.46)	0.250
War Affected Access							
No	Reference	179 (34.9)	62 (27.3)	1.00 (Reference)	—	1.00 (Reference)	—
Yes		334 (65.1)	165 (72.7)	1.43 (1.02–2.02)	0.040*	1.09 (0.72–1.66)	0.680

OR, odds ratio; CI, confidence interval. ^†^History of eye disease in the past year. ^‡^Adjusted for all variables listed in the table using multivariable logistic regression. Comprehensive screening was defined as receiving a dilated fundus examination within the preceding 12 months. * Indicates statistical significance at p < 0.05.

### Factors associated with good knowledge

To identify variables independently associated with the continuous knowledge score regarding diabetic retinopathy, we conducted a multiple linear regression analysis. The final multivariable model demonstrated a strong fit, explaining 43.2% of the variance in patients’ knowledge scores (Adjusted R² = 0.432, F = 30.56, p < 0.001). In the adjusted model, socioeconomic factors specifically education, residence, and income emerged as the strongest correlates of awareness. Educational attainment showed a profound, graded association with knowledge; compared to participants with tertiary education (18–24 years), those with primary (5–9 years) (β = -2.43; 95% CI: -3.00 to -1.86; p < 0.001) and preparatory education (10–12 years) (β = -2.20; 95% CI: -2.73 to -1.68; p < 0.001) demonstrated markedly lower overall knowledge scores. Similarly, participants with secondary education scored significantly lower compared to those with tertiary education (β = -0.89; 95% CI: -1.31 to -0.47; p < 0.001). Geographic disparities were also evident, with rural residents scoring significantly lower than their urban counterparts (β = -0.87; 95% CI: -1.27 to -0.48; p < 0.001). Furthermore, economic instability appeared to hinder awareness, as participants with a low monthly income (insufficient for basic needs) had significantly lower knowledge scores compared to those with a middle income (β = -1.02; 95% CI: -1.48 to -0.57; p < 0.001).

Regarding clinical characteristics and contextual barriers, experiential knowledge played a critical role; participants who had no previous history of eye disease scored significantly lower on the knowledge assessment (β = -1.25; 95% CI: -1.64 to -0.86; p < 0.001) compared to those who had previously experienced eye disease. Additionally, the absence of comorbid hypertension was associated with lower disease-specific knowledge (β = -0.74; 95% CI: -1.14 to -0.33; p < 0.001). Crucially, when evaluating the role of the ongoing crisis, reporting that the conflict adversely affected healthcare access emerged as a strong independent correlate of higher health literacy; affected patients scored, on average, 1.02 points higher than unaffected patients (β = 1.02; 95% CI: 0.64 to 1.40; p < 0.001). Notably, variables that exhibited severe multicollinearity or small sub-categories (specifically Occupation and Type of Diabetes) were purposefully excluded from this final model to maintain mathematical stability (VIF < 5). Full linear regression results are presented in **Table 6**.

### Factors associated with good practice

To identify factors independently associated with screening adherence, a multivariable binary logistic regression analysis was conducted, with “good practice” strictly defined as receiving a comprehensive dilated fundus examination in the preceding 12 months. In the adjusted multivariable model, educational attainment remained a significant factor, though primarily for the lowest education bracket. Participants with only primary education demonstrated significantly lower odds of receiving a dilated exam compared to those with tertiary education (AOR = 0.46; 95% CI: 0.24–0.86; p = 0.020). Geographic location profoundly influenced practice patterns, with rural residents showing significantly reduced odds of undergoing a dilated examination compared to urban residents (AOR = 0.42; 95% CI: 0.27–0.66; p < 0.001). Among clinical characteristics, a history of ocular complications demonstrate a strong association with adherence; participants without a history of eye disease were significantly less likely to engage in comprehensive screening (AOR = 0.56; 95% CI: 0.36–0.85; p = 0.010) compared to those with known eye disease.

Crucially, when assessing the association with the ongoing crisis, reporting that the conflict adversely affected healthcare access did not emerge as independently associated with screening adherence (AOR = 1.09; 95% CI: 0.72–1.66; p = 0.680). Notably, while male sex, single marital status, low monthly income, and the absence of hypertension showed significant associations in the crude analysis, these factors did not retain statistical significance after adjustment for confounders (p > 0.05). This suggests that geographic structural barriers (rural residence), severe educational disparities (primary education only), and baseline disease awareness (previous eye history) are the primary, overriding drivers of comprehensive health-seeking behavior in this population.

## Discussion

This study provides a comprehensive assessment of knowledge and screening practices related to diabetic retinopathy among adults with diabetes in Palestine during a period marked by substantial healthcare system disruption. The findings suggest that, although general awareness of diabetes-related eye complications is high, important gaps remain in the practical knowledge needed to support preventive behavior and appropriate screening. In addition, while many participants reported contact with eye-care services, substantially fewer reported receiving a dilated fundus examination within the preceding year, indicating a gap between general eye examination and appropriate retinal screening. Multivariable analyses further showed that lower educational attainment, rural residence, and absence of prior eye disease were the strongest independent correlates of poorer comprehensive screening adherence, whereas conflict-related healthcare disruption was associated with higher knowledge but not with improved screening practice. Collectively, these findings point to a disconnect between awareness, practical understanding, and the ability to access effective preventive eye care.

The present findings reveal a clear distinction between general awareness of diabetes-related eye disease and more specific understanding of diabetic retinopathy. This pattern does not appear to be unique to our study. Similar gaps have been reported in other patient populations from low- and middle-income settings, including Ethiopia, where awareness that diabetes can affect the eyes was higher than knowledge of diabetic retinopathy itself, its risk factors, and appropriate eye-care practices (2, 7). More broadly, international literature suggests that patients often retain general messages about diabetes-related eye complications but may not receive, understand, or recall the more practical information needed to act on asymptomatic disease risk (8, 9). At the same time, these findings should not be interpreted as implying that patients are expected to master detailed retinal microvascular pathology or specialist ophthalmic terminology. It would be unreasonable to expect most lay people to describe diabetic retinopathy in highly technical terms or to know all treatment modalities in detail. However, it is clinically important that people with diabetes understand several practical points: that diabetic retinopathy may develop without symptoms, that regular retinal screening is needed even when vision appears normal, that systemic factors such as glycemic control and hypertension influence risk, and that early detection and treatment can reduce the risk of visual loss (8–10). From this perspective, low awareness of diabetic retinopathy as a retinal complication, limited recognition of some important risk factors, and uncertainty regarding available treatments remain meaningful findings because they may reduce the perceived urgency of screening among asymptomatic individuals.

Notably, despite high acknowledgment of the need for eye examinations among individuals with diabetes, understanding of the appropriate timing and purpose of screening was incomplete. A substantial proportion of participants did not identify immediate screening following diabetes diagnosis as necessary, reflecting potential misconceptions regarding the asymptomatic nature of early-stage diabetic retinopathy. Comparable gaps between general awareness and practical screening knowledge have also been reported in other settings, including mixed-methods work from higher-resource contexts, suggesting that this problem is not limited to LMICs alone (8, 9). Our study did not assess what education patients with diabetes routinely receive in Palestine at diagnosis or during follow-up, and therefore we cannot determine whether the observed knowledge gaps reflect limited counseling, inconsistent delivery of health education, or incomplete patient recall. Accordingly, the statement that “current educational efforts” may be insufficient should be interpreted cautiously: the present study identifies a knowledge gap, but it does not directly evaluate the structure or content of existing educational programs in Palestine.

The findings of this study indicate that, while engagement with eye care among individuals with diabetes is relatively common, receipt of comprehensive retinal screening remains limited. Most participants reported having undergone at least one eye examination since their diabetes diagnosis; however, only 30.7% reported a dilated fundus examination within the preceding 12 months, falling short of international recommendations for regular retinal screening among people with diabetes (8, 10). Although direct comparison should be interpreted cautiously because studies differ in design, case definition, and clinical setting, this proportion appears lower than that reported in some hospital-based LMIC studies, including the Debark study in Northwest Ethiopia (7). More broadly, international evidence suggests that diabetic retinopathy screening uptake across LMICs remains variable but often suboptimal, with persistent barriers related to cost, access, low perceived need, and weak referral pathways (9, 11). Taken together, our findings suggest that the observed screening rate in this Palestinian cohort is concerning and appears, at least when compared with some published LMIC studies, to be worse rather than comparable.

Importantly, the reported content of eye examinations suggests a discrepancy between perceived and actual diabetic retinopathy screening. Only a minority of participants confirmed undergoing a dilated fundus examination, whereas a considerable proportion reported vision testing alone. In this sense, the quality of screening remains suboptimal, not necessarily because patients never attend eye care, but because those encounters do not consistently appear to include the examination most relevant to diabetic retinopathy detection. Visual acuity assessment without retinal evaluation is insufficient for detecting early diabetic retinopathy, which is frequently asymptomatic (10). Similar gaps between eye-care contact and appropriate retinal screening, as well as similar barriers related to poor communication and low perceived need, have been reported internationally (9–11). These findings suggest that some individuals may incorrectly equate routine vision assessment with comprehensive retinal screening, potentially resulting in false reassurance and delayed diagnosis.

Barriers to screening were not limited to access-related constraints. Among participants who did not undergo eye examination within the past year, the most frequently cited reason was the perception that screening was unnecessary, followed by lack of physician recommendation and financial limitations. These findings are consistent with international literature showing that low perceived need, lack of provider advice, financial burden, and logistical difficulty are recurring barriers to diabetic retinopathy screening in both LMICs and underserved populations more broadly (9, 11). This has important implications for responsibility and accountability in screening uptake. Adherence to guideline-recommended screening intervals should not be viewed solely as the responsibility of the patient. Patients require understandable information and motivation to attend, but the healthcare system and providers also have a clear responsibility to identify eligible patients, recommend screening, create referral pathways, and ensure that retinal assessment is distinguished from routine vision testing. Thus, improving uptake requires both patient engagement and health-system support.

The multivariable analyses identified educational attainment, place of residence, and history of eye disease as the variables most strongly associated with comprehensive screening practices. These findings are broadly in keeping with reports from other LMIC settings rather than representing a substantially different pattern. Studies from Ethiopia and broader international literature have similarly shown that lower educational attainment, rural residence, and barriers related to access and health literacy are associated with poorer uptake of diabetic retinopathy screening or regular eye examinations (6, 7, 9). In our study, only primary education remained significantly associated with poorer screening adherence after adjustment, suggesting that the effect of education on practice may be most pronounced at the lowest level of formal schooling. Likewise, the strong negative association with rural residence is consistent with evidence that geographic barriers, travel burden, and limited access to specialist services remain important determinants of preventive eye-care use in resource-constrained settings (12–14). The association between prior eye disease and greater adherence also supports a pattern seen in other settings, in which engagement with eye care often occurs reactively after ocular symptoms or diagnosis rather than proactively through routine preventive screening (15).

Rural residence emerged as independently associated with lower knowledge scores and reduced comprehensive screening adherence, even after adjustment for sociodemographic and clinical factors. This association likely reflects structural barriers, including limited availability of ophthalmology services, transportation difficulties, and reduced access to specialist referral pathways. Similar geographic disparities have been documented internationally and are a recurring feature of inequitable access to preventive eye care (12–14). Income level was also associated with knowledge outcomes, indicating that socioeconomic status may influence access to health information and preventive services. Financial constraints may further compound barriers to screening through direct costs, opportunity costs, or competing priorities, particularly in resource-limited settings. These findings are consistent with broader evidence demonstrating the complex relationship between social determinants and engagement with preventive healthcare (14).

Notably, a history of eye disease demonstrated one of the strongest associations with both knowledge and screening adherence. Participants without prior eye disease were substantially less likely to demonstrate high knowledge scores or to undergo comprehensive screening, suggesting that engagement with eye care often occurs reactively, following the onset of ocular symptoms or diagnosis, rather than through proactive preventive screening. This pattern is not unique to our cohort and is consistent with evidence from other settings showing that previous interaction with eye services often increases awareness and follow-up behavior (7, 15). Within the Palestinian context, these educational deficiencies coexist with a high clinical disease burden; Shrateh et al. (16) previously reported that longer diabetes duration, physical inactivity, and poor glycemic control are strongly associated with diabetic retinopathy among Palestinian adults with type 2 diabetes. The coexistence of a recognized clinical risk profile with limited disease-specific understanding underscores the need for interventions that link diabetes follow-up more directly with preventive eye care.

Contextualizing our findings within comparable regional and international settings reveals important nuances in preventive eye-care utilization. While our cohort demonstrated relatively high overall eye clinic contact, our strict definition of comprehensive screening exposed a substantial quality gap. Specifically, only 30.7% of our participants reported a dilated examination, despite much higher general eye examination uptake. This discrepancy highlights that general engagement with eye clinics does not inherently ensure comprehensive diabetic retinopathy screening and suggests systemic gaps in both screening quality and patient comprehension. These patient-level gaps may be compounded by provider-level barriers. In our cohort, absent physician advice was a frequently cited barrier to screening, a finding that parallels deficiencies documented among primary healthcare providers in other settings, including Saudi Arabia and Indonesia (3, 4). The convergence of these findings across diverse settings reinforces that inadequate integration of diabetic retinopathy education and referral into primary care is a broader systems-level challenge rather than a problem unique to Palestine.

The conflict-related findings require cautious interpretation. Our multivariable analysis revealed that reporting conflict-related impairment in healthcare access was associated with higher disease-specific knowledge, but not with improved comprehensive screening adherence. Direct comparison with findings from other countries is limited because relatively few studies have specifically examined diabetic retinopathy screening behavior in active conflict settings. However, broader literature from underserved populations and from studies addressing continuity of chronic disease care during humanitarian disruption suggests that heightened health concern may coexist with unchanged or reduced service utilization when mobility, cost, insecurity, and service continuity are compromised (15, 17, 18). Our findings appear consistent with this broader pattern. In other words, conflict may increase perceived vulnerability and motivation to monitor health, but it does not remove the structural barriers required to obtain preventive retinal screening. Thus, baseline inequities related to geography, education, and previous connection to eye care may remain more influential determinants of screening behavior than conflict-related disruption alone.

These findings carry important implications for clinical practice and public health planning. Because general utilization of eye-care services does not reliably ensure appropriate retinal screening (10), interventions must target both disease-specific understanding and the organization of care. Healthcare providers, particularly in primary care and endocrinology, should integrate diabetic retinopathy education into routine diabetes management and clearly distinguish the need for a regular dilated fundus examination from routine vision testing (8, 13, 19). Structured, disease-specific education should be interpreted in practical rather than highly technical terms. In the Palestinian context, such education could feasibly be delivered through primary care clinics, diabetes follow-up services, ophthalmology clinics, diabetes nurses, and other healthcare workers involved in chronic disease management. Brief standardized counseling, Arabic patient information materials, checklist-based referral prompts, reminder systems, and closer communication between diabetes services and eye-care providers are realistic interventions that may improve screening uptake (8, 13, 19).

From a health-systems perspective, overcoming entrenched socioeconomic and geographic disparities, which may be further exacerbated during periods of conflict, requires equitable, low-burden service delivery models. The lower adherence observed among rural and less-educated populations highlights the need to expand access through mobile screening units, community-based retinal photography, and tele-ophthalmology (14, 19). Finally, the reported conflict-related barriers underscore the importance of incorporating chronic disease prevention into emergency and humanitarian planning. Flexible service delivery and continuity-oriented screening models may help preserve preventive eye care, mitigate avoidable vision loss, and protect health equity among vulnerable populations during periods of instability (18).

This study has several notable strengths. The large sample size, which exceeded the calculated minimum requirement, enhances the precision and robustness of the findings. The inclusion of participants recruited through both clinical settings and online platforms allowed for broader coverage of individuals with diabetes and increased the diversity of the study population. In addition, the use of multivariable regression analyses enabled identification of independent factors associated with diabetic retinopathy-related knowledge and screening practices, providing insight into socioeconomic and geographic disparities. A further strength is the incorporation of conflict-related factors, which offers valuable contextual understanding of how healthcare disruption relates to preventive eye-care behaviors in a setting affected by prolonged instability.

Several limitations should be considered when interpreting the results. The cross-sectional design precludes causal inference between knowledge, screening practices, and associated factors. Data on eye examinations, screening behaviors, and clinical parameters, specifically HbA1c values, were self-reported and may be subject to recall bias or social desirability bias, particularly regarding the type of eye examination received and the accuracy of historical glycemic control. The hybrid sampling approach, including clinic-based recruitment and online snowball sampling, may limit the generalizability of findings to the broader population of adults with diabetes. In addition, although we strictly defined comprehensive screening as receiving a dilated fundus examination, misclassification of this outcome remains possible given potential patient confusion between routine vision testing and true retinal evaluation. Furthermore, the present study did not assess the content or consistency of diabetic retinopathy education received by participants at diagnosis or during follow-up, limiting our ability to interpret the observed knowledge gaps in relation to existing educational practices. Finally, some categorical variables contained small sample sizes, which may have led to wider confidence intervals for selected subgroups, while other variables had to be excluded from the multivariable models to maintain statistical stability.

Future research should address these limitations by employing longitudinal study designs to better assess causal relationships between knowledge, health behaviors, and screening outcomes. Validation of self-reported screening practices through clinical records or retinal imaging data would improve accuracy and allow clearer differentiation between comprehensive retinal screening and non-specific eye examinations. Further studies should also explore the effectiveness of targeted educational interventions and alternative screening delivery models, such as tele-ophthalmology and community-based retinal photography, particularly in rural and conflict-affected settings. Qualitative research involving both patients and healthcare providers may provide deeper insight into barriers to diabetic retinopathy screening, the content of educational messages currently delivered in practice, and the feasibility of integrating diabetic retinopathy prevention more effectively into routine diabetes care.

## Conclusion

This study shows that, although general awareness of diabetes-related eye disease is high among adults with diabetes, clinically meaningful knowledge and guideline-concordant diabetic retinopathy screening remain insufficient. While many participants reported regular eye examinations, comprehensive screening through dilated fundus examination was less consistently achieved, suggesting critical gaps in screening quality and patient understanding of what constitutes appropriate diabetic retinopathy screening. Severe educational disparities, rural residence, and a lack of prior eye disease emerged as the overriding factors associated with poor screening adherence, outweighing acute financial constraints. Crucially, while conflict-related healthcare disruptions paradoxically increased disease-specific knowledge and the desire to monitor health, these heightened attitudes did not translate into increased comprehensive screening adherence. This underscores a profound divergence between crisis-induced health awareness and the physical ability to overcome entrenched geographic and structural inequities to access preventive eye care during instability.

## Data Availability

The raw data supporting the conclusions of this article will be made available by the authors, without undue reservation.
